# How accurately can other people infer your thoughts—And does culture matter?

**DOI:** 10.1371/journal.pone.0187586

**Published:** 2017-11-07

**Authors:** Constantinos Valanides, Elizabeth Sheppard, Peter Mitchell

**Affiliations:** University of Nottingham, School of Psychology, University Park, Nottingham, United Kingdom; George Mason University, UNITED STATES

## Abstract

This research investigated how accurately people infer what others are thinking after observing a brief sample of their behaviour and whether culture/similarity is a relevant factor. Target participants (14 British and 14 Mediterraneans) were cued to think about either positive or negative events they had experienced. Subsequently, perceiver participants (16 British and 16 Mediterraneans) watched videos of the targets thinking about these things. Perceivers (both groups) were significantly accurate in judging when targets had been cued to think of something positive versus something negative, indicating notable inferential ability. Additionally, Mediterranean perceivers were better than British perceivers in making such inferences, irrespective of nationality of the targets, something that was statistically accounted for by corresponding group differences in levels of independently measured collectivism. The results point to the need for further research to investigate the possibility that being reared in a collectivist culture fosters ability in interpreting others’ behaviour.

## Introduction

The purpose of this research was to investigate whether people can determine what others are thinking (something positive or something negative) after observing a brief sample of their behaviour. A secondary aim was to begin to investigate a potentially relevant factor to success in this arena–cultural background. In the following paragraphs, we define ‘mentalizing’ and explain why cultural background could be a factor that is worthy of investigation.

‘Mentalizing’ (known otherwise as mindreading, theory of mind, mental simulation, mind perception, empathic accuracy) is inferring others’ mental states, usually for the purpose of explaining past behaviour or predicting future behaviour [1]. One research tradition has focused on the developmental origins and the cognitive process involved in mentalizing [2] while another has focused on devising techniques intended to determine how accurately any given individual can infer others’ mental states [3]. Inspired by the desire to unite these two research traditions [4], we employ a technique designed to investigate a particular aspect of mentalizing called ‘retrodictive mindreading’ [5, 6], which is interpreting another person’s behaviour to infer the proximal cause (an underlying mental state) and the distal cause (the event that evoked the mental state—perhaps something that happened externally in the world). The research technique is notable in that it satisfies West and Kenny’s [7] ‘truth condition’. These authors identify a major problem with past research into mentalizing concerning Person A (the perceiver) making an inference of Person B’s (the target’s) mental state: Because we the researchers do not truly know the target’s mental state it is therefore difficult to evaluate the status of the perceiver’s inference. West and Kenny also argued that judgments of mental states are subject to bias and that it is important at least to measure this bias so that it can be separated from other aspects of performance.

The task we employ is adapted from a technique used previously [6, 8, 9, 10, 11, 12, 13, 14, 15]. An event causes targets to have a mental experience, an experience which is naturally expressed and signalled in aspects of visible behaviour, including their facial expressions. Video recordings of the target’s signalled mental state are shown to perceivers whose task is effectively to infer the event that caused the target’s mental state. The perceiver’s response can then be compared against an objective fact that is known to the researcher (and that is independent of the perceiver’s judgment and separate from any declaration from the target)–that is, the researcher knows independently and for a fact which of various events the target truly experienced and he or she is thus able to determine categorially whether the perceiver’s inference is correct or incorrect.

In addition to satisfying West and Kenny’s ‘truth condition’, this task also offers a further benefit. The reaction of the target, which is video recorded without their knowledge, is completely spontaneous and natural (it is not posed, contrived or enacted). Because this task satisfies the truth condition, perhaps it offers advantages over a widely used procedure for investigating ‘empathic accuracy’ in relation to natural and spontaneous reactions, developed by Ickes and colleagues [3]. In that task, two people, a target and a perceiver, engage in conversation while being videoed. Later, the target watches the video of themselves and notes what they were thinking or how they felt at any given moment. Meanwhile, the perceiver also watches the video of the target and estimates what they (the target) were thinking and how they were feeling. If the perceiver’s estimation corresponds with the target’s declarations, then the perceiver is adjudged to have a high level of empathic accuracy, meaning effectively that the perceiver has read the target’s mind.

In this task, though, we cannot be sure that the target’s declaration of their mental state is a reliable and valid source of information, in which case the truth condition is not satisfied. We cannot be sure that the target’s mind is sufficiently transparent to itself such that the target can simply report their own mental states; even if it were, we cannot be sure that the target will be sufficiently eloquent to articulate their mental states; even if they were, we cannot be sure that the target would choose to be honest in their reporting.

Still, if the perceiver’s estimate of the target’s mental state corresponds with the target’ declaration, would that provide the necessary reassurance that the perceiver can indeed read the target’s mind? Not necessarily. If the target’s mind were not transparent to itself, then on viewing a video of themselves presumably they would resort to interpreting clues in their own behaviour that are visible in the video, as they would in interpreting the behaviour of a third party. In other words, targets and perceivers would effectively be undertaking the same task, namely to interpret clues in the behaviour of a person they observe in a video; and it is hardly surprising to find, then, that their judgments concur–but this should not be taken as a sign that the perceiver can determine from observing the target’s behaviour something that the target knows independently through introspection. Hence, there is no basis for supposing that Ickes’ procedure satisfies the truth condition and in the light of this we prefer the retrodictive mentalizing task.

One purpose of the current research is to extend the scope of the retrodictive mindreading procedure. In past research [11], the behaviour of the experimenter, particularly something she said, provoked a reaction in targets that perceivers were subsequently able to interpret–they could guess what the experimenter had said to the target or what kind of gift she had offered. Despite the many virtues of such a method, a skeptic might argue that when perceivers guessed what caused the target’s reaction, they bypassed the mind of the target and made a direct connection between the target reaction and the event in the world. To avoid such criticism, in the current research target behaviour was not in reaction to some or other event but rather was an external manifestation of internal target thoughts of a past event. Specifically, targets were cued to think of a past event that caused them to experience a specified emotion. Hence, perceivers in the current research were effectively being asked to infer what targets were thinking, not what they were reacting to. Would perceivers be able to guess what the targets had been cued to think when the target’s behaviour was an external manifestation of internal thoughts (reflections on a past event) and was not in response to the unexpected or unusual behaviour of a third party? If so, this would offer more compelling evidence for mindreading (of a mental state signalled in the target’s behaviour) in a condition that nevertheless satisfies the truth condition—for we the researchers know independently and as a matter of fact which cue word had been presented to the target.

In conducting this research we are also exploring whether people whom we might suppose were raised in a collectivist culture (in this case, people from southern European states) are better at mentalizing than those whom we might assume were raised in an individualistic culture (British people). Here, we define collectivism as being more concerned with others, sharing material resources, and being attuned to the implications for others of their decisions [16]. Granted, there are regional variations within cultures [17], and while on average it is reasonable to suppose that collectivism prevails in southern European states while individualism prevails in Britain [18, 19, 20, 21], it is nevertheless essential to independently verify levels of collectivism and individualism [22]. Notwithstanding, on the face of things, it might seem implausible to consider that collectivists will be better at mentalizing given that some past research points to tardy development in the ability to impute a false belief among children growing up in collectivist cultures [23, 24, 25]. It does not necessarily follow, however, that prolonged development leads ultimately to under-development.

According to Nisbett [25], an individualistic culture promotes the development of an independent mode of thinking in which independence and self-reliance are emphasized [26]. A collectivist culture, by contrast, promotes an interdependent mode of thinking revolving around group membership and interpersonal relationships. Because people reared in collectivist cultures are encouraged to think more about others compared with people reared in individualistic cultures, they are effectively being nurtured to assume commonality between their own and other minds [27] and accordingly become attuned to other minds in so far as they are adapted to use their own mind to model the mental states being experienced by other people [28]. Mitchell et al [22] thus investigated whether people reared in Mediterranean states, who were identified by an independent measure as being collectivist, display superior skills to British participants (who were identified independently as being individualistic) in imputing mental states to others. In the task, participants observed a scenario in which a protagonist saw an object in Location A but later heard a message saying it was in Location B. In one condition, but not another, participants (but not the protagonist) had additional privileged information that the message was true. Participants then judged where the protagonist believed the object was. British participants were biased to judge that the protagonist believed what they themselves believed, suggesting their own knowledge egocentrically contaminated their estimation of what a third party believed [29]. In contrast, Mediterranean participants were more likely to judge that the protagonist would believe the message, irrespective of any privileged information they, the participants, had received uniquely. Hence, the collectivist Mediterranean participants exhibited less egocentric bias than the individualistic British participants and in consequence it is tempting to conclude that they might be better equipped to make accurate estimations of what others are thinking.

Clearly, there are limitations in drawing such a conclusion. First, the results revealed a difference between participant groups in biased reasoning, not directly in accurate mentalizing. According to West and Kenny [7], it is vital to separate bias from accuracy when investigating how well people mentalize. Second, the task does not satisfy West and Kenny’s truth condition, for we do not know what the protagonist really thinks. Third, the task is highly contrived in that participants were presented with a stylised hypothetical scenario which did not involve interpretation of spontaneous target behaviour.

In addition to comparing the mentalizing ability of collectivist Mediterraneans and individualistic British people, the methods used also enable us to test the possibility of an in-group advantage in mentalizing [30]: An advantage in imputing mental states to members of one’s own social or cultural group. Previous research suggests that expressions of emotion may differ across cultures due to differing display rules [31]. If this is the case, then we may find participants are better at interpreting the behaviour (and perceiving the minds) of individuals from their own than from another culture. At the very least, then, it will be useful to measure the level of expressiveness of targets (participants who are cued to think of something from the past), independently of measures of how well observers (perceivers) can infer what the targets are thinking.

## Method

### Overview

Two kinds of participant were recruited, targets and perceivers. In the stimulus development phase, targets were cued by one of four words (pride, excitement, shame, guilt) appearing on a laptop screen to think for thirty seconds about a time in their life when they experienced the named emotion very intensely. As they did so, they were surreptitiously filmed by the laptop’s webcam. In the main experimental phase, perceivers watched the videos of the targets and were invited to guess which word was being displayed on the screen in front of the target. Half of the targets were British and half were Mediterranean; likewise, half of the perceivers were British and half were Mediterranean, though none of the perceivers were given any explicit information to indicate that the targets they viewed were from two distinct cultural backgrounds. Participants also filled in a questionnaire that was designed to identify individual differences in collectivism and individualism. The entire procedure was approved by the Ethics Committee, School of Psychology, University of Nottingham, and all participants (targets and perceivers) who provided useable data gave written informed consent.

Participants (targets and perceivers) completed a questionnaire [32] whose aim was to identify individual differences in collectivism and individualism. The questionnaire included thirty-six questions that formed six subscales. Three subscales probed collectivism and another three probed individualism. The subscales of collectivism were the sense of common in-group fate (six questions), familialism (seven questions) and interrelatedness (six questions). The subscales of individualism included valuing personal uniqueness (six questions), valuing personal freedom and happiness (five questions) and valuing personal achievement (six questions).

#### Targets–stimulus development phase

Twenty-eight targets were recruited (British: 2 males and 12 females, Mediterranean: 5 males and 9 females) aged between 18 and 32 (M = 23.7, SD = 3.5), all university students residing in the UK. The 14 Mediterranean targets were born and raised in Greece (7) and Cyprus (7), states which are known for their collectivist culture [14, 19, 20, 21]. A further target was also tested but he or she did not give consent for his/her video to be used in the main experimental phase of the study.

#### Materials and apparatus–stimulus development phase

**Fig 1:**
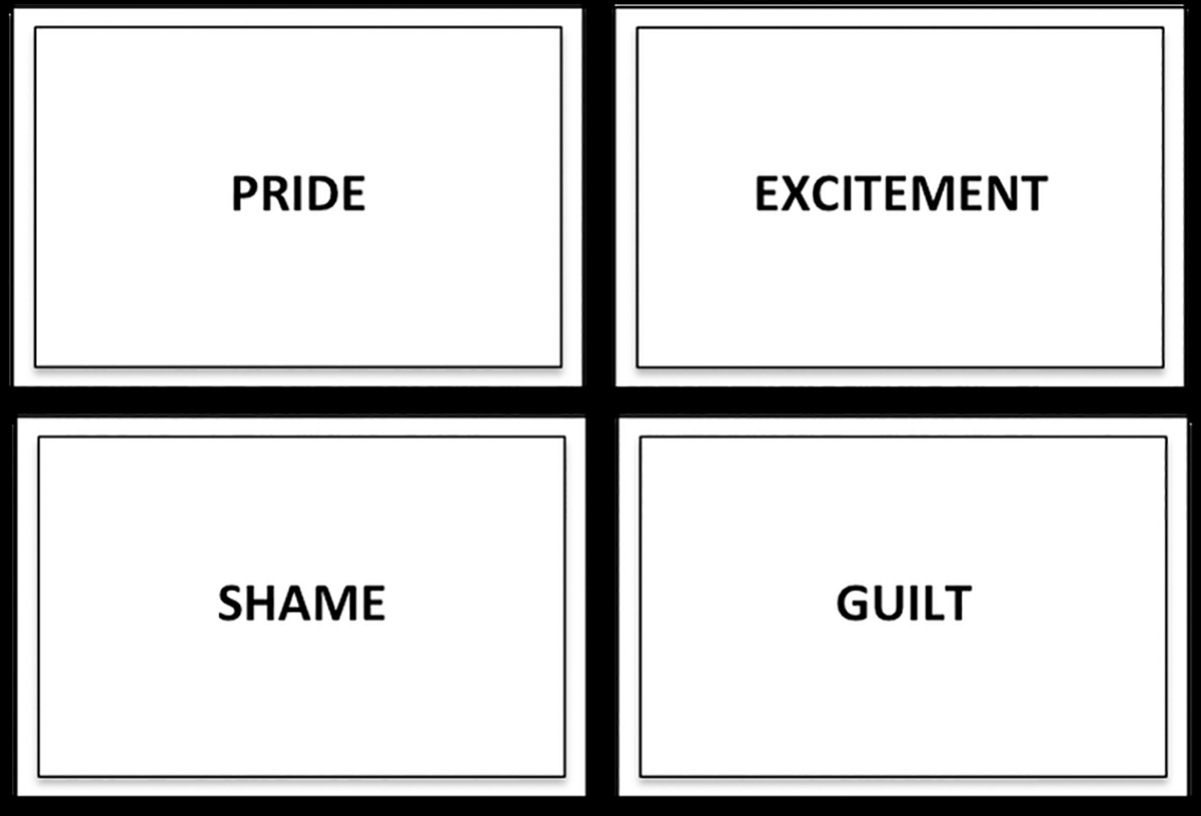
Cue words presented to targets. Only one word, framed as shown in the figure, appeared (in the centre of the laptop screen) at any one time.

The words shown in Fig 1 were presented to the targets using PsychoPy [33] in the middle of a Toshiba laptop screen. The size of each label was 1024 by 768 pixels. Meanwhile, the laptop’s webcam surreptitiously filmed the targets (the light was disabled that would otherwise indicate recording) as they were thinking about the experiences that led to the labelled emotions. The targets sat approximately 0.70 meters from the screen such that their face and shoulders were recorded.

#### Procedure–stimulus development phase

The experiment was carried out in an unoccupied, spacious and quiet room. Prior to starting, targets were briefed that they would be asked to think about a situation in the past that caused them to feel pride, excitement, shame and guilt. They were informed that they did not have to describe this situation–just think about it. Furthermore, as a decoy, targets were told that they would later be asked to explain how difficult it had been for them to retrieve those memories for each emotion. The true aim of the experiment, however, was to record the facial expression and body language of the target while they were thinking about the event that caused them to feel each particular emotion.

The order of the labelled emotions was counterbalanced and targets either began with two positive emotions (pride, excitement) or the two negative emotions (shame, guilt). Before the experiment began, the experimenter left the room so that the target was alone and would not feel inhibited by the presence of another person. Targets read a screen of brief instructions that ended by asking them to press ‘space’ to begin the task. A short tone (four seconds) to prepare the target preceded the appearance of each labelled emotion (Fig 1). Targets then had thirty seconds to think about the situation that caused them to have the labelled emotion. Prior to leaving the testing room targets were debriefed about the true purpose of the study which was to investigate if other people (perceivers) could guess what they were thinking after watching a video of them as they were recalling an event that caused them to experience a named emotion. They were also informed that the reason why they were asked to retrieve those memories (some of which might have been uncomfortable) was to elicit a reaction within a short period of time.

The video recordings of targets were edited in VLC Media Player (Version 2.2.1) [34], and Windows Live Movie Maker and VirtualDub (Version 1.9.11) [35]. VLC Media Player was used to crop the targets’ 30-second videos, one for each emotion. Windows Live Movie Maker and VirtualDub were used to remove the sound from the videos. Thus, four videos were collected from each target. A total of 112 videos (28 targets each generating 4 videos) were available for use in the main experimental phase.

#### Perceivers–main experimental phase

Thirty-two participants (17 males and 15 females) aged between 22 and 32 (M = 24.4, SD = 2.5), all university students residing in the UK, served as perceivers. Sixteen (10 males and 6 females) were British and 16 (7 males and 9 females, 10 Greek–Cypriots, 3 Greeks, 1 Turkish, 1 Spanish and 1 Italian) were Mediterranean born and bred in nations that are known for their collectivist culture [14, 19, 20, 21]. Each received £5 as an inconvenience allowance for one hour of their time.

#### Procedure–main experimental phase

Perceivers were tested in a quiet room individually. Prior to starting the experimental task they completed the questionnaire. Perceivers were briefed that they would be watching video clips of people thinking about past events, cued by the words *pride*, *excitement*, *shame* and *guilt*. Perceivers were told that they would be asked to guess which cue word the target was looking at and therefore which kind of past event they were thinking about. Subsequently, each perceiver viewed all 112 videos (1424 by 950 pixels), each appearing for 30 seconds, on a Toshiba laptop screen in randomised order using Psychopy. Perceivers sat approximately 0.70 meters from the screen. After each video ended, a screen with the four emotion words appeared and perceivers had to press a key to register their response (1 = pride, 2 = excitement, 3 = shame, 4 = guilt). Psychopy recorded the perceiver’s choice along with their response time measured from the point when the choices appeared on the screen to the point at which they pressed a key. Hence, response time included reading time, though note that options had the same content and appeared in the same fixed order in every trial, thus removing the burden of reading new material on successive trials. The main experimental phase of the procedure occupied approximately one hour.

## Results

### Questionnaire analysis

Raw data appear in S1 Data. Initially, questionnaire results for targets and perceivers were combined. We used the Cronbach test to determine if participants answered questions consistently within the six sub-scales [36]. The three subscales for collectivism were reliable, with Cronbach *α* = 0.81, 0.90, 0.62 for common fate, familialism and interrelatedness respectively for British people, and Cronbach *α* = 0.83, 0.73, 0.61 for common fate, familialism and interrelatedness respectively for Mediterranean people. The three subscales for individualism were reliable for British people with Cronbach *α* = 0.87, 0.66, 0.89 for personal uniqueness, personal freedom/happiness and personal achievement respectively. In contrast, only the personal achievement subscale was reliable for Mediterraneans, with Cronbach *α* = 0.72. The subscales for personal uniqueness and personal freedom/happiness were unreliable with Cronbach *α* = 0.54 and 0.46, respectively. The average score for each subscale (common fate, familialism and interrelatedness) was measured first. Then the collectivism score for each culture-group was calculated based on the average score of their three subscales. The average collectivism score for British was *μ* = 2.49 and for Mediterraneans was *μ* = 2.77. Mediterraneans thus achieved higher collectivism scores than British people, *t*(58) = -2.69, *p* = 0.01.The scores for the personal achievement subscale, the only individualism scale that consistently achieved an acceptable level of reliability, were not significantly different between the two cultures, *t*(58) = -0.99, *p* = 0.09.

### Main analysis

The main purpose of the study was to determine how well perceivers inferred which emotion cue word was being displayed to targets as they were thinking of an event in their life that caused them to experience the displayed emotion. Preliminary analysis revealed two things. First, perceivers were unable to discriminate between target expressions of the same valence. In other words, for example, there was no evidence to suggest that perceivers selected ‘shame’ more often on the occasions when targets were cued to think of an event that caused them to feel shame than on the occasions when cued to think of an event that caused them to feel guilty (*t*(15) = 1.98, *p* = 0.07, for British perceivers and *t*(15) = 0.24, *p* = 0.82, for Mediterranean perceivers); and neither were they able to discriminate between targets thinking of pride and targets thinking of excitement (*t*(15) = 1.07, *p* = 0.30, for British perceivers and *t*(15) = 0.69, *p* = 0.49, for Mediterranean perceivers). Accordingly, we focused on perceivers’ ability to discriminate between targets thinking of something positive (pride, excitement) and targets thinking of something negative (shame, guilt). Second, perceivers were biased in judging whether targets were thinking of something positive or negative. We used an odds ratio procedure where the value of 1 indicates that ascriptions of positive thoughts presented an equal number of times as ascriptions of negative thoughts. A one sample *t*-test with *μ* = 1 revealed that the odds ratio value was significantly different from 1 for British perceivers, *t*(15) = 5.77, *p* = 0.001, as well as for Mediterranean perceivers, *t*(15) = 3.43, *p* = 0.004. The mean odds ratio did not significantly differ between the two groups, *t*(30) = 0.40, *p* = 0.69. Both groups of perceivers were thus biased to judge that targets were thinking of something negative and there was no evidence to suggest that the two groups differed in the level of bias.

**Fig 2:**
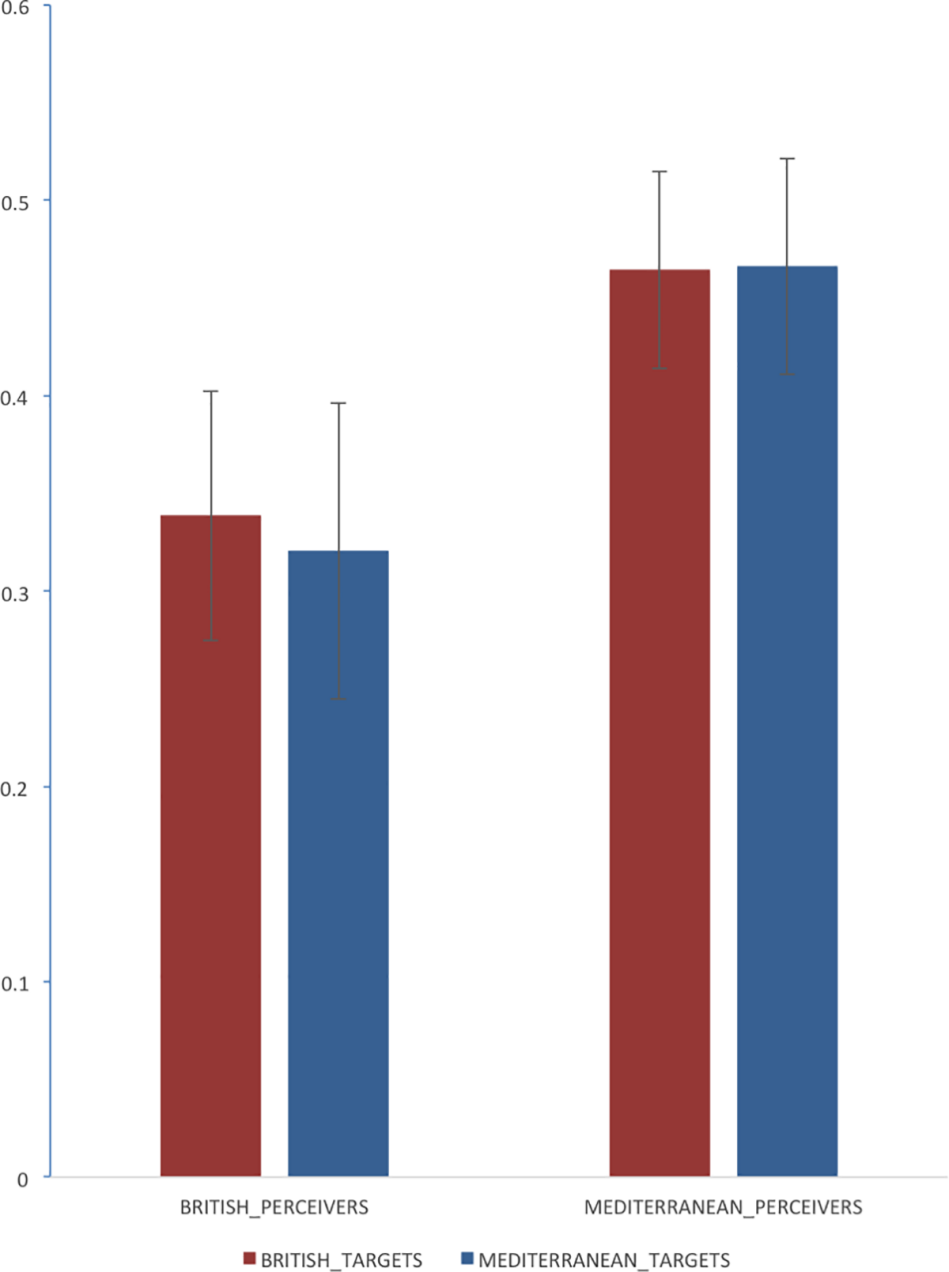
Mean *d’* scores. British and Mediterranean perceivers made judgments on whether British and Mediterranean targets were thinking of something positive or something negative. Standard errors of the mean are represented by the error bars.

We classified perceivers’ responses as ascribing a positive thought (irrespective of whether pride or excitement) and ascribing a negative thought (irrespective of whether shame or guilt). These were coded as hits (e.g. ascribing a positive thought when the target was viewing a positive emotion cue word), false alarms (e.g. ascribing a positive thought when the target was viewing a negative emotion cue word), misses (e.g. ascribing a negative thought when the target was viewing a positive emotion cue word) and correct rejections (e.g. ascribing a negative thought when the target was viewing a negative emotion cue word). This provided sufficient information to calculate two d-prime (*d'*) values for each perceiver, indexing their ability to discriminate between conditions in which targets were thinking of something positive or something negative, corrected for bias to judge that targets were generally thinking of something negative. One d-prime value represented the perceiver’s sensitivity to what Mediterranean targets were thinking and the other represented the perceiver’s sensitivity to what British targets were thinking. If any *d'* value is equal to zero, it would mean that perceivers were insensitive to whether targets were thinking of something positive or negative. Fig 2 crosses the nationality of the target with the nationality of the perceiver, yielding four mean *d'* values. All values are greater than zero according to one-sample *t*-tests (*p* < .001 in all cases). Hence, perceivers were systematically able to determine whether targets were thinking of something positive or something negative. In answer to the primary research question, it seems that perceivers were effective to some degree in interpreting the behaviour of the targets to infer what they were thinking.

Moving on to the secondary research question, the pattern of means depicted in Fig 2 seems to suggest that Mediterranean perceivers were better at inferring target thoughts than British perceivers. A 2 (perceiver nationality) X 2 (target nationality) analysis of variance (ANOVA), with the last factor being a repeated measure, was conducted on perceivers’ *d'* values. A large main effect [37] confirmed that Mediterranean perceivers were indeed better at estimating target thoughts than British perceivers were, *F*(1,30) = 4.81, *p* = 0.03,*η*^*2*^ = 0.14. The main effect associated with target nationality and the interaction term were nonsignificant (*F*<1 in both cases).

The Mediterranean perceivers may have been better than British perceivers in estimating target thoughts because of their higher level of collectivism. This is a plausible suggestion on finding that (1) Mediterranean perceivers had higher collectivism scores than British perceivers (*p* = 0.02) and (2) perceivers who were better at estimating what targets were thinking (indicated by higher *d’* values) also tended to have higher collectivism scores, *r* = 0.86, *n* = 32, *p*<0.001. Using multiple regression, we modelled variance that represented how well perceivers could estimate what targets were thinking (*d’* values) using culture group and collectivism scores as predictors, *R*^*2*^ = 0.74. The inclusion or exclusion of the variable culture group made no significant difference to the amount of variance accounted for, suggesting that more accurate inferences in Mediterranean perceivers was associated with (and perhaps explained by) higher levels of collectivism in those perceivers.

### Response times

Making correct inferences about target thoughts might require time and effort. If so, those who performed especially well and gained high *d'* scores should have taken longer in total time to complete the task than those with lower *d'* scores. Contrary to this expectation, perceivers with the highest *d'* scores completed the task in the least time, *r* = -0.55, n = 32, *p* = 0.001. The results of a 2 (nationality of perceiver) X 2 (nationality of target) X 2 (type of thought experienced by target) ANOVA, the last two factors being repeated measures, performed on response time data, yielded no significant results (*F*<1 in all cases).

### Supplementary study—target expressiveness

Despite the absence of evidence to suggest any cultural variations among targets in how easily perceivers can infer what they are thinking, it is still legitimate to enquire whether some target groups were more expressive than others. Triandis [26] reports that collectivistic groups can be more expressive than individualistic groups. Also, some evidence suggests that females can be more emotionally expressive than men [38]. However, it does not necessarily follow that it is easier to infer the inner states of targets who are more expressive than those who are less expressive [13]. That is, a high level of expressivity does not necessarily give a clearer signal of inner states.

To investigate target expressiveness, we recruited 10 British nationals of Northern European descent (5 males; 5 females), aged 20 to 47 years (M = 27.6, SD = 8.0). They viewed all 112 target videos (28 targets, each cued by 4 different emotion words) and rated how expressive the target was in each video on a 6-point scale (1 = least expressive, 6 = most expressive). As with the study reported above, the perceivers viewed the videos on a laptop using PsychoPy from a distance of about 0.7M; the rating scale appeared beneath each video. We thus calculated the mean of four expressivity values (averaging across the four cued emotions) for each target, based on the ratings of the 10 perceivers and raw data appear in S2 Data. British female targets were rated on average as 2.60 (SD, .60) on a scale of expressivity from 1–6, where 6 is high. British males were rated as 3.26 (SD, .68), Mediterranean females were rated as 2.60 (SD, .74) and Mediterranean males were rated as 2.40 (SD, .31). We performed a 2 (target gender) X 2 (target nationality) between-groups ANOVA on these data. None of the main effects and none of the interaction terms was significant, giving no evidence to suggest, in this case, that targets varied in their level of expressivity depending on their gender or depending on their nationality.

## Discussion

Even though perceivers (irrespective of nationality) were biased in ascribing negative emotions to targets, they were nevertheless sensitive to whether targets were thinking of something positive or something negative. In addition, Mediterranean perceivers were more accurate than British perceivers in determining whether targets were thinking of something positive or something negative (irrespective of target nationality). The advantage apparent in Mediterranean perceivers in determining target thoughts was accounted for by their higher levels of collectivism, compared with British perceivers. Perceivers who were good at detecting target emotions also took less time to complete the task compared with those who were not good, but this did not manifest as an average speed advantage in Mediterranean perceivers compared with British perceivers. And there was no evidence to suggest that levels of expressivity among different target groups impacted upon perceiver sensitivity to what targets were thinking.

The evidence was strong and consistent in showing that perceivers were correctly able to infer whether targets were thinking of something positive or something negative, though they were not able to discriminate one positive state from another (pride and excitement) and neither were they able to discriminate one negative state from another (shame and guilt). It thus seems that perceivers are effective in detecting broad attitude domains of thought (positive or negative) but not subtle distinctions within a domain. As far as mentalizing is concerned, then, there is no evidence to suggest that perceivers would be able to interpret the precise content of thought in a target although they can determine broadly whether target thoughts are positive or negative.

Compared with past research that employs a similar procedure [11], the results reported here seem to demonstrate for the first time that perceivers can indeed perform a mentalisitc interpretation of target behaviour when that behaviour is triggered by internal reflections. The finding reported here thus helps to address a potential criticism of past research, namely that it is questionable whether perceivers were reading the minds of targets; instead, perhaps they were merely making a direct link between target behaviour and an external event while bypassing any consideration of the target’s mind. Accordingly one might question the degree to which perceivers in previous research carried out a kind of processing that deserves to be called ‘retrodictive mindreading’ [5].

In the current research, the target’s behaviour was an external manifestation of their inner reflections, not a reaction to a contemporary external event. Hence, perceivers in this research were indeed tasked with inferring what targets were thinking in such a way that does not allow a skeptic to say that perceivers were directly linking target behaviour with an external event but without considering what the target was thinking. Admittedly, one might argue that perceivers were associating target behaviour with a *previous* external event (for example, the episode, whatever it was, that caused the target to feel pride), but such a suggestion would have to suppose that target behaviour in reaction to an event is the same as target behaviour when recalling that event, and there is no evidence for such a supposition [39]. Consequently, the demonstration of retrodictive mindreading is perhaps much more compelling in the current research compared with previous research.

Having established that perceivers were at least able to determine whether targets were thinking of something positive or something negative, it was then legitimate to address the secondary question of whether some groups of perceivers perform more accurately than others. In this context, one might reason that being reared in a collectivist culture fosters attunement to other minds because such a culture, unlike an individualistic culture, promotes perception that other minds are similar to one’s own. Accordingly, collectivist perceivers might be inclined to use one’s own mind as a model for mental simulation [28]. Despite this reasoned basis for expecting better performance in Mediterraneans than British, past circumstantial evidence has been either inconsistent or insufficient to lend compelling support to the suggestion. Tardy development in acquiring the ability to pass a test of false belief [23, 24] might have been taken as a sign that people reared in a collectivist culture have rather poor aptitude for mentalizing. In principle, though, it does not necessarily follow that prolonged development implies inferior development. If that were so, then humans, a species noted for protracted development, would also be rather incompetent relative to those species who develop rapidly. Hence, all things equal, slow development might actually lead to a better outcome as far as mentalizing ability is concerned.

Although past research was suggestive of more accurate mentalizing in Mediterraneans than British [22], the evidence in question was insufficient. The findings merely demonstrated that Mediterraneans were more inclined than British to judge that a protagonist would be credulous and believe what he was told. This bias, if it deserves to be called such, seemed to protect Mediterraneans from conflating their own belief with the protagonist’s but it does not necessarily follow that Mediterraneans are more accurate in mentalizing over and above any immunity to bias that might affect performance. In the current research bias was evident in judgments made by perceivers–namely, they were biased to ascribe negative thoughts to targets. Unlike in the research by Mitchell et al, however, this bias is not confused with evidence that speaks to good performance in mentalizing. That is, the results suggest Mediterraneans were more accurate in mentalizing irrespective of any measurable bias. Hence, the findings presented here perhaps offer the first demonstration that Mediterraneans are more accurate than British in this sphere of mental activity.

Why were Mediterraneans better? The research took place in the UK with British participants who had not ventured abroad (at least not in this instance) and Mediterraneans who had. Is it therefore possible that Mediterranean perceivers performed well because the special characteristics associated with studying abroad (such as being outward looking, mental strength, sense of adventure) are also associated with good skills in mentalizing? While we cannot completely rule out this possibility, it is worth noting that the better performance of Mediterraneans was entirely statistically explained by their higher collectivism scores as measured by a self-report instrument. It thus seems that Mediterraneans were more accurate than British in mentalizing because of their higher levels of collectivism (and perhaps not because of any other factor associated with studying abroad).

In what sense were Mediterraneans more accurate than British in mentalizing? Perhaps working out what a target is thinking requires time and effort rather than talent. In other words, is it possible that Mediterraneans gave more correct judgments because they were conscientiously spending more time? An analysis of response times offered no evidence to suggest that Mediterraneans took longer than British perceivers to complete the task. Nevertheless, it is still legitimate to enquire whether those perceivers who achieved high mentalizing scores also spent longer on the task, irrespective of nationality. The data provide a very clear answer to this question, which is that, on the contrary, those who achieved highest scores tended to complete the task in the least time. The relationship between speed and accuracy does not reveal a trade-off between the two [40] but rather suggests that people who have the talent to give a correct mentalizing judgment are able to do so quickly. Hence, the results testify to competence rather than effort in this arena.

The design used in this study allowed us to determine whether perceivers were more easily able to infer what Mediterranean targets than what British targets were thinking. If Mediterraneans are more expressive than British People [26], then perhaps this elevated level of expressiveness would provide an interpretable signal to perceivers that reveals what the target is thinking. By analogy, it seems that the behaviour of typically developing targets contains a much clearer signal to perceivers than the behaviour of people with autism, meaning that perceivers can infer mental states in those who are typically developing much more accurately than in those who have autism [13, 39]. However, the current findings offer no evidence to suggest that perceivers are more accurate in mentalizing about Mediterranean targets than British targets. Besides, the supplementary data we collected from a separate group of perceivers provided no evidence to suggest that the Mediterranean targets were indeed more expressive than the British targets in this case.

The design used in this study also allowed us to determine how perceivers perform in inferring mental states in people of the same and different backgrounds. Past research shows that perceivers are better at processing the faces of targets who belong to the same rather than different groups [30, 31, 41, 42]. From this, it might follow that perceivers are sufficiently effective in processing the faces of targets belonging to the same group as themselves such that they are advantaged when inferring mental states. However, there was no evidence in our data to suggest that a perceiver-target similarity effect confers any benefit in mentalizing.

In conclusion, the results demonstrate people’s ability to infer what others are thinking. While past research tells us that perceivers can guess what a third party said or did to a target, the current research goes further in showing that perceivers can infer whether the target is thinking of something positive or negative in a circumstance where these thoughts are not caused by a contemporary external event but by internal musings. Targets’ inner thoughts thus gave rise to an outward expression in the form of a signal that was readily interpretable by perceivers. The results also raise the possibility that a collectivist culture promotes the aptitude to interpret inner states that are signalled in the expression of a target. Perhaps the acquisition of such aptitude is explained by the value placed on thinking about other minds as being similar to one’s own that prevails in collectivist cultures [26], something that could nurture sensitivity to the meaning of signals inherent in others’ behaviour. That said, the current research is limited by relatively small sample size and by differences in participant groups according to whether or not they were studying abroad. The current findings encourage further research to test the robustness of cultural differences in accurate inferences of inner states.

## Supporting information

S1 Data Raw data for main study. A spreadsheet of data that analyses were conducted upon for the main empirical study.
(CSV)

S2 Data Raw data for supplementary study. A spreadsheet of data that analyses were conducted upon for the supplementary empirical study.
(XLSX)
